# Glomerular solidification is associated with nephritis-related clinical parameters in IgA nephropathy

**DOI:** 10.1080/0886022X.2019.1665545

**Published:** 2019-09-26

**Authors:** Tsutomu Inoue, Yankun Luo, Takeru Seto, Hiromichi Suzuki, Hirokazu Okada

**Affiliations:** a Department of Nephrology, Faculty of Medicine, Saitama Medical University, Saitama, Japan;; b Department of Nephrology, Shanxi Provincial People’s Hospital, Taiyuan, China;; c Musashino Tokusyukai Hospital, Tokyo, Japan

**Keywords:** Glomerulosclerosis, IgA nephropathy, obsolescence, solidification

## Abstract

**Background:** Two types of global glomerulosclerosis, glomerular obsolescence and solidification, have been identified. A clinicopathological correlation between these glomerular changes and hypertensive nephrosclerosis has been reported; however, clinicopathological correlations with other kidney diseases are unknown. The aim of this study was to evaluate the correlation between the two glomerulosclerosis types and the clinical IgA nephropathy presentation.

**Methods:** A single center, cross-sectional study of patients with IgA nephropathy was performed. Correlations between glomerulosclerosis and body mass index, mean blood pressure, creatinine-based estimated glomerular filtration rate (eGFR), total cholesterol, urinary protein corrected by urinary creatinine, and anti-hypertensive agent use were investigated using univariate and multivariate analyses.

**Results:** Overall, 116 patients were enrolled (male/female, 59/57; mean age, 40.5 ± 15.0 years). Separate analyses were performed for solidification and obsolescence glomerulosclerosis. Univariate analysis demonstrated a significant correlation between the percentage of solidification glomerulosclerosis and patient age, mean blood pressure, eGFR, and use of antihypertensive drugs. Multivariate analysis showed that only eGFR and use of antihypertensive drugs maintained their independent predictive value. The amount of urinary protein emerged as a significant factor based on the multivariate analysis. However, although the univariate analysis demonstrated a statistically significant correlation between the percentage of obsolescence and eGFR for obsolescence glomerulosclerosis, a multivariate analysis indicated that none of the factors maintained their independent predictive value.

**Conclusions:** The incidence of solidification was better correlated with some nephritis-related clinical parameters compared with the incidence of obsolescence. The emergence of solidification may influence the clinical activities that are associated with IgA nephropathy.

## Introduction

IgA nephropathy is the most prevalent primary chronic glomerular disease worldwide. It is defined immunohistochemically by a predominance of IgA deposits, either alone or in conjunction with IgG, IgM, or both, in the glomerular mesangium [1]. Histological findings range from a virtually normal appearance by light microscopy to severe necrotizing, crescentic glomerulonephritis, or advanced glomerulosclerosis and tubular atrophy with interstitial fibrosis. Similar to the histological heterogeneity observed, the clinical course of IgA nephropathy is also variable. There is a common understanding that some patients with IgA nephropathy will show a progressive course and have a poor prognosis, which will eventually reach end-stage renal disease (ESRD). Several histological lesions have been reported to be of prognostic value. Generally, reports have shown that, where the clinical outcome is the time to ESRD/dialysis, chronic lesions, including tubular atrophy, interstitial fibrosis, and glomerulosclerosis, are the most important histological predictors [2,3]. Global glomerulosclerosis correlates well with tubulointerstitial alterations, and the presence of lesions has been proposed to be a poor prognostic factor, although interstitial fibrosis and tubular atrophy were stronger predictors compared with global glomerulosclerosis when the Oxford classification was used [2,3] because quantification is less susceptible to errors resulting from the presence of few glomeruli.

Two types of glomerular sclerosis, glomerular obsolescence and solidification, were originally described in the 1960s. Since then, differences in the features of obsolescent and solidified glomerulosclerosis have been reported in hypertensive nephrosclerosis by Marcantoni et al. [4,5]. These previous reports stated that these two types of glomerulosclerosis were not consecutive features of a unique glomerular pathology, but that they originated from different pathogenic mechanisms. These studies also observed solidified glomeruli significantly more often in African-Americans compared with Caucasians, which might be explained by excessive autoregulatory responses in susceptible patients, such as African-Americans. However, there was no significant difference in the percentage of obsolescent glomeruli between African-Americans and Caucasians. Obsolescent glomeruli probably reflect a milder mechanism of injury that is possibly associated with aging [4]. The percentage of obsolescent glomeruli increases with age, while that of solidified glomeruli does not increase with age. Obsolescent glomeruli are considered to be a consequence of ischemia that is caused by a narrowing of intrarenal vessels, whereas solidified glomeruli are thought to be associated with excessive autoregulatory responses and genetic factors.

These two types of global glomerulosclerosis have completely different appearances and seem to be induced by different pathogenic mechanisms. Clinicopathological correlations between these glomerular changes and hypertensive nephrosclerosis have been reported; however, clinicopathological correlations in other kidney diseases, such as IgA nephropathy, remain to be clarified. In this study, we observed obsolescent and solidified glomerulosclerosis in IgA nephropathy biopsies and we evaluated potential clinical pathological correlations.

## Methods

### Study design and subjects

**Figure 1.**
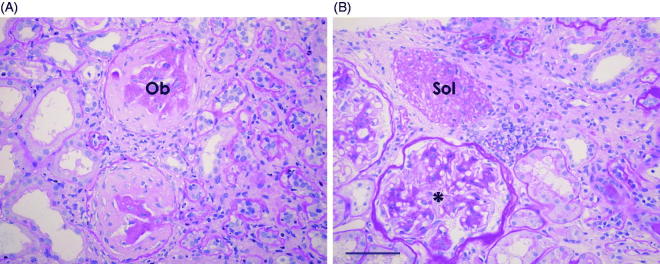
Representative histological findings in renal biopsies from IgA nephropathy patients. Ob in (A) indicates obsolescent glomerulus; Sol in (B) indicates a solidified glomerulus observed with periodic acid-Schiff stain. * in (B) indicates a glomerulus with marked mesangial proliferation. Scale bar in (B) = 50 μm.

This was a single center, cross-sectional, observational study. Subjects were enrolled for 5 years from October 2009 until September 2014. During this period, 400 renal biopsies were performed and patients aged ≥16 years with biopsy-proven IgA nephropathy (*n* = 116) were enrolled. The diagnosis was made using light microscopy and immunofluorescence. Patients for whom the biopsy included fewer than 10 glomeruli were excluded from the analysis. All biopsy specimens with periodic acid-Schiff (PAS) staining were independently reevaluated by the authors YL and HO. The presence of the two types of global glomerulosclerosis was assessed and quantified as a percentage of the total number of glomeruli for each biopsy. Results were obtained as the average of the data from both YL and HO. Before histologic evaluation of IgA nephropathy, several hypertensive nephrosclerosis tissue specimens were used to confirm mutual recognition of the characteristics of two types of global glomerulosclerosis. The method described above was based on a previous report [5]. Solidified glomeruli were defined as glomeruli in which the entire tuft was solidified in the absence of collagenous changes in the capsular space. This can be described by the term ‘decompensated benign nephrosclerosis.’ Obsolescent glomeruli were defined as glomeruli in which the Bowman’s space was occupied by collagenous, PAS-positive material, and the tuft was retracted. Figure 1 shows representative findings for two types of global glomerulosclerosis in IgA nephropathy in our patients.

Clinical information was obtained from electronic health records and data for the following parameters at the time of admission for renal biopsy were evaluated: body mass index (BMI; kg/m^2^) = body weight/body height^2^; mean blood pressure (MBP; mmHg) = diastolic pressure + (systolic pressure – diastolic pressure)/3; creatinine-based estimated glomerular filtration rate (eGFR) calculated using the Modification of Diet in Renal Disease (MDRD) formula for Japanese [6]; eGFR (mL/min/1.73 m^2^) = 194 × Cr^−1.094^ × Age^−0.287^ (×0.739 for female patients); total cholesterol (mg/dL), urinary protein corrected by the urinary creatinine (g/1.0 g urinary creatinine); and the use of any anti-hypertensive agents.

### Ethics approval and consent to participate

This study is based on only existing information, such as clinical data without subject invasion or intervention, and on the guidelines for research in Japan. Information on the full scope of the study, such as subjects, purpose, and survey content, must be made public and opportunity for refusal must be granted in place of obtaining written informed consent. The institutional review board (IRB) at our institution, in accordance with the Declaration of Helsinki and the Japanese guidelines for clinical research, approved this research and its publication. This manuscript was originally designed in 2015, but there was a subsequent revision of the guidelines in Japan in 2017. We reevaluated our research plan and method based on these new guidelines, and the study now complies with the new guidelines and was approved by our IRB (Approval No. 17–033-1 and 14–060-1).

### Statistical analysis

Initially, univariate linear regression was used to assess the associations between the percentage of solidified glomeruli or the percentage of obsolescent glomeruli (dependent variables) and each clinical parameter (independent variables). A multivariate linear regression analysis was then performed to avoid overestimation of potentially linked variables. Multicollinearity was evaluated by the variance inflation factor. To compare two groups, Fisher’s exact test or the Wilcoxon signed-rank test were used. JMP version 10.0 (SAS Institute Inc., Cary, NC, USA) and EZR version 1.20 (R Development Core Team, Vienna, Austria; and the modified R commander for medical research in Japan) [7] software programs were used for all statistical analyses. The data are presented as the mean ± standard deviation unless otherwise specified. A value of *p* < 0.05 was considered statistically significant.

## Results

The patient characteristics at biopsy are summarized in Table 1. Among the 48 patients who were using antihypertensive drugs, 42 patients (87.5%) were treated with an angiotensin-converting enzyme inhibitor (ACE-I) and an angiotensin receptor blocker (ARB). We also independently examined the background of the subjects with obsolescence, solidification, or both types of global sclerosis. The usage rate of antihypertensive drugs in the ‘Only Solidified glomeruli’ group was significantly higher than in the ‘Only Obsolescent glomeruli’ group; however, other factors were similar. This suggests that patients with solidified glomeruli showed more proteinuria and/or hypertension compared with patients with obsolescent glomeruli (Table 2).

**Table 1.** Summary of the clinicopathological parameters of study subjects.

	Values
Number of Cases	116
Gender, Male, *n* (%)	59 (50.9)
Age (years)	40.5 ± 15.0
Body Mass Index	23.8 ± 3.8
Mean Blood Pressure (mmHg)	95.7 ± 14.2
Estimated Glomerular Filtration Rate (mL/min/1.73m^2^)	77.6 ± 27.7
Total Cholesterol (mg/dL)	224.0 ± 68.3
Urinary Protein (g/g·uCr)	2.1 ± 3.5
Treatment with any Antihypertensive Drug(s), *n* (%)	48 (41.4)
% of Glomerular Solidification (% Sol)	6.3 ± 10.7
% of Glomerular Obsolescence (% Ob)	4.1 ± 8.5

uCr: urinary creatinine; Sol: Glomerular Solidification; Ob: Glomerular Obsolescence.

**Table 2.** Summary of the clinicopathological parameters grouped according to glomerular sclerosis type.

	Total	Without any Glomerular sclerosis	Only Solidified glomeruli	Only Obsolescent glomeruli	With both Glomerular sclerosis
Number of Cases	116	46	27	24	19
Gender, Male, *n* (%)	59 (50.9)	26 (56.5)	14 (51.9)	11 (45.8)	8 (42.1)
Age (years)	40.5 ± 15.0	34.7 ± 14.8	44.6 ± 13.4	45.2 ± 16.3	40.5 ± 15.4
Body Mass Index	23.8 ± 3.8	22.9 ± 4.2	23.1 ± 3.3	23.0 ± 3.3	23.7 ± 4.0
Mean Blood Pressure (mmHg)	95.7 ± 14.2	93.4 ± 14.2	99.7 ± 14.6	93.7 ± 12.6	98.2 ± 14.8
Estimated Glomerular Filtration Rate (mL/min/1.73m^2^)	77.6 ± 27.7	82.3 ± 27.8	77.8 ± 27.0	77.1 ± 32.0	66.4 ± 20.9
Total Cholesterol (mg/dL)	224.0 ± 68.3	226.1 ± 78.7	222.8 ± 44.7	238.6 ± 84.9	200.8 ± 30.3
Urinary Protein (g/g・uCr)	2.1 ± 3.5	2.3 ± 3.9	1.5 ± 1.6	3.1 ± 5.2	1.2 ± 0.9
Treatment with any Antihypertensive Drug(s), *n* (%)	48 (41.4)	11 (23.9)	18 (66.7)*	8 (33.3)	11 (57.9)
(Treatment with ACE-I/ARB), *n* (%)	42 (36.2)	10 (21.7)	16 (59.3)*	7 (29.2)	9 (47.4)

uCr: urinary creatinine; ACE-I: angiotensin-converting enzyme inhibitor; ARB: angiotensin receptor blocker. Asterisks indicate statistically significant differences between the “Only Solidified glomeruli” and “Only Obsolescent glomeruli” groups by Fisher’s exact test.

The univariate analysis demonstrated a statistically significant correlation between the percentage of solidified glomeruli and patient age, mean blood pressure, eGFR, and use of antihypertensive drugs (*p* = 0.016, 0.042, 0.002, and 0.001, respectively). A multivariate analysis showed that only eGFR and the use of antihypertensive drugs maintained their independent predictive value. The amount of urinary protein emerged as an important factor based on the multivariate analysis for solidified glomeruli. The whole model, including eGFR, urinary protein, and use of antihypertensive drugs, was also a significant predictive model for the percentage of solidified glomeruli (adjusted *R*^2^ = 0.19, *p* = 0.0001; Table 2). However, a multivariate analysis showed that none of the factors maintained their independent predictive value, although the univariate analysis demonstrated a statistically significant correlation between the percentage of obsolescent glomeruli and eGFR (*p* = 0.022; Table 3).

**Table 3.** Summary of univariate and multivariate linear regression analyses.

	Solidified glomeruli					Obsolescent glomeruli				
	Regression Coefficient	SE	Standardized Regression Coefficient	*t*	*p*-Value	Regression Coefficient	SE	Standardized Regression Coefficient	*t*	*p*-Value
Simple linear regression										
Gender, Male	−0.959	0.997	−0.090	−0.960	0.338	−1.218	0.783	−0.144	−1.560	0.122
Age	0.161	0.065	0.224	2.460	0.016*	0.077	0.052	0.136	1.470	0.144
Body Mass Index	0.192	0.265	0.068	0.720	0.471	0.056	0.210	0.025	0.270	0.789
Mean Blood Pressure	0.143	0.070	0.189	2.060	0.042*	0.063	0.056	0.106	1.140	0.257
Estimated Glomerular Filtration Rate	−0.111	0.035	−0.287	−3.190	0.002*	−0.065	0.028	−0.212	−2.320	0.022*
Total Cholesterol	−0.009	0.015	−0.061	−0.620	0.537	0.002	0.010	0.024	0.240	0.811
Urinary Protein	−0.289	0.287	−0.094	−1.010	0.316	0.026	0.227	0.011	0.110	0.909
Treatment with any Antihypertensive Drug	3.360	0.966	0.310	3.480	0.001*	−0.358	0.802	−0.042	−0.450	0.656
Multivariate linear regression										
Gender, Male	−0.707	0.970	−0.069	−0.730	0.468	0.048	0.698	0.007	0.070	0.946
Age	−0.040	0.077	−0.058	−0.520	0.606	0.020	0.055	0.045	0.370	0.711
Body Mass Index	−0.005	0.255	−0.002	−0.020	0.985	0.083	0.184	0.048	0.450	0.651
Mean Blood Pressure	0.068	0.073	0.095	0.920	0.358	−0.009	0.053	−0.019	−0.170	0.866
Estimated Glomerular Filtration Rate	−0.127	0.044	−0.349	−2.910	0.005*	−0.055	0.031	−0.229	−1.740	0.085
Total Cholesterol	0.003	0.017	0.020	0.180	0.860	−0.001	0.012	−0.006	−0.050	0.960
Urinary Protein	−0.748	0.340	−0.266	−2.200	0.030*	−0.105	0.245	−0.057	−0.430	0.668
Treatment with any Antihypertensive Drug	2.940	0.960	0.284	3.060	0.003*	−0.088	0.690	−0.013	−0.130	0.898
	*p* = 0.002; *R*^2^ = 0.22; Adjusted *R*^2^ = 0.15					*p* = 0.671; *R*^2^ = 0.06; Adjusted *R*^2^ = −0.02				
Estimated Glomerular Filtration Rate	−0.144	0.036	−0.373	−4.010	0.000*					
Urinary Protein	−0.754	0.284	−0.246	−2.650	0.009*					
Treatment with any Antihypertensive Drug	3.055	0.916	0.281	3.330	0.001*					
	*p* < 0.0001 ; *R*^2^ = 0.22 ; Adjusted *R*^2^ = 0.19									

SE: standard error.

## Discussion

In this study, two types of global glomerulosclerosis were associated with IgA nephropathy, as was previously reported for hypertensive nephrosclerosis [4]. Additionally, solidified glomeruli, but not obsolescent glomeruli, were correlated with the clinical severity of IgA nephropathy, as were eGFR and the presence of hypertension. The results of statistical analyses indicated that both types of glomerulosclerosis had completely different properties, although their incidence was similar, in the biopsy specimens examined (Table 1). This suggests that different mechanisms may contribute to the development of these lesions, as previously reported [4].

In the present study, all subjects were Japanese; therefore, differences in genetic background between subjects are unlikely to account for the differences observed. Similar to hypertensive nephrosclerosis, it is well known that global glomerulosclerosis in IgA nephropathy is also significantly correlated with segmental sclerosis and tubulointerstitial damage [2,3]. Although no previous studies have investigated which type of global sclerosis is responsible for the poor prognosis, solidified glomeruli appear to be involved based on our present results. It is difficult to evaluate the relationship between solidified glomeruli and tubulointerstitial damage in our patients, because their tubulointerstitial damage was slight. However, a correlation between the degree of interstitial damage and remaining renal function, and the significant relationship between eGFR and percentage of solidified glomeruli demonstrated in the current study support a relationship between solidified glomeruli and tubulointerstitial damage. Additionally, segmental glomerulosclerosis is an initial glomerular lesion that is associated with disease progression in hypertensive nephrosclerosis and IgA nephropathy [3].

Previous studies reported a significant correlation between solidified glomeruli and segmental glomerulosclerosis/tubulointerstitial alterations [4,5]. An imbalance in the glomerular perfusion pressure results in segmental sclerosis, which is followed by the solidified type of global glomerulosclerosis with tubulointerstitial alterations. The strong correlation between interstitial fibrosis and solidified glomeruli in hypertensive nephrosclerosis supports this hypothesis [1]. Segmental glomerulosclerosis can be caused by various mechanisms. IgA nephropathy is commonly considered to result from segmental podocyte detachment. The initial mechanisms underlying the formation of segmental sclerosis are different from those hypertensive nephrosclerosis, where segmental sclerosis causes the adaptation and segmental resistance to collapse and reflow. It is possible that the attendant segmental hypertension and hyperfiltration could give rise to the progression of segmental sclerosis, resulting in glomerular solidification in IgA nephropathy, as well as hypertensive nephrosclerosis. ACE-I and ARB reduced proteinuria and slowed disease progression in patients with hypertensive nephrosclerosis and IgA nephropathy [8,9]. This suggests that the underlying mechanisms of progression have a commonality.

This study had some limitations. It was a single center design and a small scale, non-longitudinal, cross-sectional study. Additionally, the patients should ideally be compared before ACEi/ARB treatment; however, medications were started before the renal biopsy was taken if the patient had shown moderate proteinuria or persistent hypertension. This point may have affected the results. The significance of assessing the two types of glomerular sclerosis separately lies in the potential to obtain a more accurate prognosis for patients with IgA nephropathy. It would be interesting to determine whether the Oxford classification together with consideration of the difference in global glomerular sclerosis is more useful than analysis without the evaluation of global glomerular sclerosis (original style) to predict the prognosis of IgA nephropathy in a longitudinal study. We are planning a catamnestic observation over the next 5–10 years to examine this possibility. The two types of sclerosed glomeruli might represent different stages of the same process, which cannot be completely ruled out. To resolve this issue, a prospective multicenter clinical study with a larger sample size that includes patients who underwent a re-biopsy is required in the future. Moreover, cystatin C-based GFR estimation may be better than using creatinine-based eGFR for this study because most subjects did not have severe renal insufficiency.

In conclusion, two types of global glomerular sclerosis, glomerular obsolescence and solidification, were observed in IgA nephropathy patients, which is similar to previous reports in patients with hypertensive nephrosclerosis. Additionally, the global sclerosis showed significantly different properties with regard to their association with clinical parameters. Based on our results, glomerular solidification might be more essential than glomerular obsolescence for the progression of IgA nephropathy. Because of the heterogeneity of the clinical course, risk assessment for the development of ESRD is necessary to consider how to balance the potential benefits and risks of treatment. It might be useful to distinguish between glomerular obsolescence and solidification for this purpose.

## Data Availability

All data underlying the findings are presented within the paper or are available upon reasonable request from the corresponding author. We will call for re-deliberation by the IRB and a collaborative research agreement will be required before providing the raw data in MS Excel format to other institutes.
